# Publisher Correction to: Journal of Intensive Care, volume 7

**DOI:** 10.1186/s40560-019-0398-9

**Published:** 2019-08-12

**Authors:** Journal of Intensive Care

**Affiliations:** London, UK

**Publisher Correction to: Journal of Intensive Care, volume 7**

An error occurred during the publication of a number of articles in *Journal of Intensive Care*. Several articles were published in volume 7 with a duplicate citation number.

In this correction article the old and new citation metadata are published in Table 1.

**Table 1:** Overview of incorrect and correct citation metadata

DOI	Incorrect citation number	Correct citation number
10.1186/s40560-019-0388-y	1	34
10.1186/s40560-019-0389-x	2	35

The original articles have been updated. The publisher apologizes for the inconvenience caused to our authors and readers.

